# Chronic Appendicitis with Report of Cases

**Published:** 1911-02

**Authors:** W. C. Cook

**Affiliations:** Columbus, Ga.


### CHRONIC APPENDICITIS WITH REPORT OF CASES.

W. C. Cook, M. D., Columbus, Ga.

So much has been written and said during the past twenty years, concerning acute appendicitis, that I will not take up your time with a discussion of that phase of the subject, but will direct my few remarks to the chronic variety of the disease, which is so protean in its manifestations that a complete analysis of the subject would be out of order here. Therefore, I will confine myself to the report of a few cases, and the conclusions which may be drawn therefrom.

Case No. 1.—Mr. B., Greek restaurant keeper, came to me about three years ago, complaining of indigestion, constipation, and a general feeling of lassitude, having had these symptoms for several years. Upon physical examination, I found an area of tenderness and soreness under the right free border of ribs, corresponding to the gall bladder region. Stomach analysis showed a very low percentage of hydrochloric acid. I made a tentative diagnosis for gall stones, and put him on the recognized medical treatment for this condition, as he would not hear to any operation. He soon drifted out of my hands, and went to first one doctor and then another, without any improvement, until, finally, about the first of February, 1910, he came back to me, complaining exacty in the same way and saying he was tired of medical treatment and wanted the operation done. Accordingly, on the 18th of February, under ether anaesthesia, I opened the abdomen through the right rectus muscle, and found the gall bladder practically normal, with no stones in it. The stomach and duodenum both seemed normal.

I now turned my attention to the appendix, although the patient had complained of no symptoms referable to that organ. Very much to my surprise the appendix was found much enlarged, and quite hard and fibrous, with several enteroliths in its lumen. The appendix was removed in the usual way, after which I opened the gall bladder and removed some thickened bile, and a small amount of bile sand, put a drainage tube in the gall bladder, and closed up the wound. He made an uninterrupted recovery. The indigestion symptoms did not all disappear at once, but they grew progressively better until at the present time he is entirely well.

Case No. 2.—Mr. J., aged about 30 years, clerk by occupation; was operated upon about 3 years ago in Augusta, Ga., for gall stones, from which he made a prompt recovery, although he had symptoms of indigestion which persisted after the operation. In November, 1909, the patient was taken with a very severe attack of intercostal neuralgia, with which he had been suffering about a month when I was called in consultation with the view of doing a neurectomy, as the process seemed to be confined to one intercostal space. Having gotten some very good results in facial neuralgia by injections of alcohol and chloroform I decided to try this treatment for a while before operating. Accordingly, I injected about 30 minims of 95% alcohol with 5 drops of chloroform into the interspace which was involved, and repeated the process in two days. For a while the pain got better, and after about three or four weeks of intermittent treatment, disappeared entirely, only to return in about another week, in another interspace and was more violent than ever, so much so that it required from 1-2 to 1 1-2 grains of morphine daily, to give him any comfort whatever. I began the injections once more and kept them up occasionally until the latter part of February. At times the pain seemed to be disappearing only to start up in a new interspace the next day, always confined to the right side. During February the worst paroxysms of pain seemed to come on after eating, the patient noticed this, and he began to abstain from eating in order to avoid as much as possible the horrible pain.

After establishing the fact that the paroxysms were always made worse by eating, I decided that the condition was either due to ulcer, or to some adhesions resulting from the former operation. I, therefore, advised an exploratory operation, which was readily agreed to. The patient was taken to the hospital, and on March 7th, under ether anaesthesia, I opened the abdomen through the old scar. The gall bladder and ducts were found absolutely normal, except that the gall bladder was adherent to abdominal wall, as was also the omentum, but the adhesions were no more than would be expected following any operation for gall stones. I then examined the stomach and duodenum, and found they were perfectly normal. On examining the appendix it was found somewhat adherent, and could not be drawn up into the high incision, accordingly the latter was enlarged downward, so as to make the removal of the appendix easier. This appendage was found very much in the same condition as in Case No. 1, enlarged, thickened, full of fecal concretions, and bound down by quite a number of adhesions. The appendix was removed, some of the worst adhesions tied off and abdomen closed. Recovery was uneventful except for a small stitch abscess, which soon healed. The neuralgic pain disappeared immediately and has never returned. Digestive disturbances disappeared gradually until now he is entirely well.

Case No. 3.—Mr. R., machinist, aged about 25. Began suffering about 3 years ago with indigestion, colicky pains in lower abdomen, and constipation. One of his most distressing symptoms was vomiting after almost every meal, as he expressed it, "I spit up nearly everything I ate." When I first saw the patient about the middle of April, 1910, he was suffering quite severe and almost continuous pain and tenderness in lower abdomen, especially on the right side. He had been passing bloody urine for the past month, so I made a cystoscopic examination, and found a small villous tumor on the base of bladder. My diagnosis was chronic recurrent appendicitis, complicated by tumor of bladder. On April 20th I opened the abdomen over McBurney's point, by means of the muscle splitting incision, and removed a very much enlarged and adherent appendix, which contained several fecal concretions. The abdomen was then closed, and I did a suprapubic cystotomy through which the bladder tumor was removed. The bladder wound was closed, and I introduced a catheter through the urethra for drainage. Convalescence was uninterrupted. The pain and tenderness disappeared at once, as did also the vomiting and other digestive disturbances. The patient is now well and about ready to return to work.

And now as to some of the conclusions to be drawn from a study of these, to my mind interesting cases. In the first place, digestive disturbance of some kind was one of the most prominent symptoms in every case. Chronic indigestion is always a symptom, and not a disease in itself to be treated independently of the cause any more than is the cough of tuberculosis to receive our entire attention in this dread disease.

In all these cases the disturbance of digestion had some of the elements of obstruction. Case three was almost typical of partial stenosis of the pylorus. Now, I think these obstructive symptoms can be accounted for as follows: The inflamed area around the head of the colon is in a crippled condition, it, therefore, cannot take care properly of the amount of food and refuse which is being passed on through the small intestine, consequently by means of the great sympathetic nerve, a telegraphic message, so to speak, is sent to the stomach not to send down so much food, hence the spasmodic effort on the part of the pylorus to prevent the passage of stomach contents, with the consequent production of symptoms. Then, again, we all know that a constant irritation of the peritoneum in any locality will retard or check the peristalic wave in the adjacent intestine, and this, I think, is the cause of the chronic constipation, which always accompanies this condition.

Another lesson is that not over half of these cases ever give symptoms directly referable to the appendix itself, but they all give symptoms of persistent indigestion. Then, again, the stomach trouble does not always disappear immediately after the operation, but grows gradually better, until, finally, it is gone entirely. Therefore, we need not be discouraged after an operation for this trouble, because the patient's digestion does not improve at once, for we may rest assured that it will do so, provided the appendix was the sole cause of the trouble.

Still another conclusion is, that very few of these cases will present any very great similarity in their symptoms. And last, but not least, it is my opinion that a large number of our cases of long-continued digestive disturbances are due to a chronic appendicitis, and having made a correct diagnosis, we may confidently assure these sufferers that a timely resort to surgery will relieve them of their trouble.

